# Intergenerational Differences in Social Media Use Among LGBTQ+ Adults

**DOI:** 10.1093/geroni/igaf122.1346

**Published:** 2025-12-31

**Authors:** Jess Francis-Levin, Bolu Dogari, Emily Briggs

**Affiliations:** University of Michigan, ANN ARBOR, Michigan, United States; University of Michigan, Ann Arbor, Michigan, United States; University of Michgian, University of Michigan, Michigan, United States

## Abstract

Older adults in who identify as LGBTQ+ are at an increased risk of becoming socially isolated. Members of the LGBTQ+ community have been shown to be innovative in their ability to create community through social media use. Although the research related to younger LGBTQ+ folks is increasing, there is still little known regarding how older adults use social media and how that compares to their younger counterparts. We explored differences in motivations to use social media among older adults (aged 55+; n = 88) and young adults (aged 18-54; n = 253) who identify as members of the LGBTQ+ community. Participants completed an online survey, rating their agreement with statements assessing their motivations for social media use on a 7-point scale. Older adults were significantly more likely to use social media for social bonding (M = 3.66) compared to younger adults (M = 3.33), Welch t (142.7) = 2.8, p = 0.005, 95% CI: (0.11, 0.60). Similarly, family requests serve as a stronger motivation for older adults (M = 2.58) than for younger adults (M = 2.32), Welch t (176.2) = 2.3, p = 0.02, 95% CI: (0.04, 0.51). These findings suggest that LGBTQ+ older adults are more likely than LBGTQ+ younger adults to use social media to maintain relationships, particularly with family members. We discuss these results further along with supporting qualitative findings from brief response survey items.

